# Chemerin Affects P_4_ and E_2_ Synthesis in the Porcine Endometrium during Early Pregnancy

**DOI:** 10.3390/ijms23020945

**Published:** 2022-01-15

**Authors:** Marlena Gudelska, Kamil Dobrzyn, Marta Kiezun, Katarzyna Kisielewska, Edyta Rytelewska, Tadeusz Kaminski, Nina Smolinska

**Affiliations:** 1 Department of Animal Anatomy and Physiology, Faculty of Biology and Biotechnology, University of Warmia and Mazury in Olsztyn, Oczapowskiego St. 1A, 10-719 Olsztyn, Poland; marlena.gudelska@uwm.edu.pl (M.G.); marta.kiezun@uwm.edu.pl (M.K.); katarzyna.kisielewska@uwm.edu.pl (K.K.); edyta.rytelewska@uwm.edu.pl (E.R.); tkam@uwm.edu.pl (T.K.); 2 Department of Zoology, Faculty of Biology and Biotechnology, University of Warmia and Mazury in Olsztyn, Oczapowskiego St. 5, 10-718 Olsztyn, Poland; kamil.dobrzyn@uwm.edu.pl

**Keywords:** chemerin, steroidogenesis, signalling pathways, endometrium, gestation, pig

## Abstract

Chemerin, belonging to the adipokine family, exhibits pleiotropic activity. We hypothesised that the adipokine could be involved in the regulation of steroidogenesis in the porcine endometrium. Thus, the aim of this study was to determine the effect of chemerin on the key steroidogenic enzyme proteins’ abundance (Western blot), as well as on P_4_ and E_2_ secretion (radioimmunoassay) by the porcine endometrium during early pregnancy and the mid-luteal phase of the oestrous cycle. Moreover, we investigated the hormone impact on Erk and Akt signalling pathway activation (Western blot). Chemerin stimulated E_2_ production on days 10 to 11 of pregnancy. On days 10 to 11 and 15 to 16 of gestation, and on days 10 to 11 of the cycle, chemerin enhanced the expression of StAR and all steroidogenic enzyme proteins. On days 12 to 13 of pregnancy, chemerin decreased StAR and most of the steroidogenic enzyme proteins’ abundance, whereas the P450_C17_ abundance was increased. On days 27 to 28 of pregnancy, chemerin increased StAR and P450_C17_ protein contents and decreased 3βHSD protein amounts. It was noted that the adipokine inhibited Erk1/2 and stimulated Akt phosphorylation. The obtained results indicate that chemerin affected P_4_ and E_2_ synthesis through the Erk1/2 and Akt signalling pathways.

## 1. Introduction

Chemerin was first described as retinoic acid responder protein 2 (RARRES2) or tazarotene-induced gene 2 protein (TIG2) [1]. Chemerin belongs to the group of adipokines, hormones produced mainly in the adipose tissue, and cytokines, a large group of proteins secreted by specific cells of the immune system [2]. The hormone is produced as a 163-amino-acid protein (prochemerin) and is non-active before the cleavage of the C-terminus [2]. Inflammation- and coagulation-related serine proteases are responsible for converting prochemerin into active forms [2]. Expression of *RARRES2*, the gene encoding chemerin, has been noted in various tissues of humans, rodents, bovine, poultry, and pigs [3,4,5,6,7]. In humans, the highest expression of the adipokine was noted in the white adipose tissue, liver, and placenta. Lower chemerin abundance was detected in the brown adipose tissue, lungs, skeletal muscles, kidneys, ovaries, and heart [8]. In the case of porcine tissues, in our previous studies, we have indicated chemerin gene and protein expression in the elements of the female hypothalamic–pituitary–gonadal (HPG) axis [4,5,6], as well as in the uterus [7].

As an adipokine, chemerin plays an important role in metabolism regulation [9]. Initially, *RARRES2* expression and circulating concentrations of the hormone are mainly transmitted with features of metabolic syndrome, such as higher plasma triglyceride levels, blood pressure, body fat content, and insulin resistance [10]. Development of type 2 diabetes mellitus has been found to highly correspond with increased chemerin levels in human serum [11]. Moreover, the hormone concentrations in the human inflammatory fluids interdepend on the concentrations of tumour necrosis factor α (TNFα), interleukin 6 (IL6), and C-reactive protein (CRP), which are the main metabolic syndrome-related factors [12]. Chemerin, as a cytokine, plays an important role in several physiological processes, such as metabolism, angiogenesis, and blood pressure control [12,13]. Chemerin’s actions are mostly associated with immune system functions. The hormone increases macrophages’ adhesion to the proteins of the extracellular matrix and induces their recruitment, chemotaxis, and retention at the inflammation site [14]. All chemerin’s actions in an organism are triggered after hormone binding to one of its three receptors: chemokine-like receptor 1 (CMKLR1), G protein-coupled receptor 1 (GPR1), and C-C chemokine receptor-like 2 (CCRL2) [8,15,16].

Recently, there have been increasing reports about the important role that chemerin might play in the reproductive system, especially in females. The participation of chemerin in the regulation of reproductive system functioning may be supported by the presence of chemerin and its receptors (collectively known as the chemerin system) in the tissues belonging to the female reproductive tract. Chemerin and CMKLR1 expression were indicated in human granulosa cells [17], whereas chemerin and GPR1 expression were noted in mouse granulosa cells, cumulus cells, and the corpus luteum (CL) [18]. Expression of the chemerin system was indicated also in the uterus of humans [19] and pigs [7], as well as in the human and rat placenta [20,21]. It was found that, in the pig, apart from the ovaries, the uterus is also a source of steroid hormones [22]. Steroids are some of the most significant factors that are involved in the maternal recognition and maintenance of pregnancy in this species [23].

The influence of hormones related to metabolism control (orexin A and B, adiponectin) on the functioning of the porcine uterus was determined in our previous studies. Both orexins and adiponectin were found to affect the gene expression of key steroidogenic enzymes (*StAR*, *CYP11A1*, *CYP17A1*, *CYP19A3*, *HSD3B1*) in the porcine uterus during early pregnancy and the mid-luteal phase of the oestrous cycle [24,25,26,27,28]. It has previously been shown that chemerin, another hormone controlling metabolism, can regulate steroidogenesis in the rat and porcine ovary [29,30]. However, there are no studies on the influence of chemerin on the process of steroidogenesis in the uterus of the domestic pig. We hypothesise that the adipokine could be a potent locally produced or adipose-derived factor involved in the regulation of steroidogenesis in the porcine endometrium. Thus, the aim of this study was to determine the effect of chemerin on progesterone (P_4_) and oestradiol (E_2_) secretion and the protein abundance of steroidogenic acute regulatory protein (StAR), P450 side-chain cleavage enzyme (P450_SCC_), cytochrome P450_C17_ (P450_C17_), cytochrome P450 aromatase (P450_AROM_), and 3β-hydroxysteroid dehydrogenase (3βHSD) in the incubated porcine endometrial tissue explants. Moreover, we wished to determine the involvement of the Erk1/2 and Akt signalling pathways in the hormone’s mechanism of action.

## 2. Results

### 2.1. The Effect of Chemerin on P_4_ and E_2_ Secretion by the Porcine Endometrial Tissue

**Figure 1:**
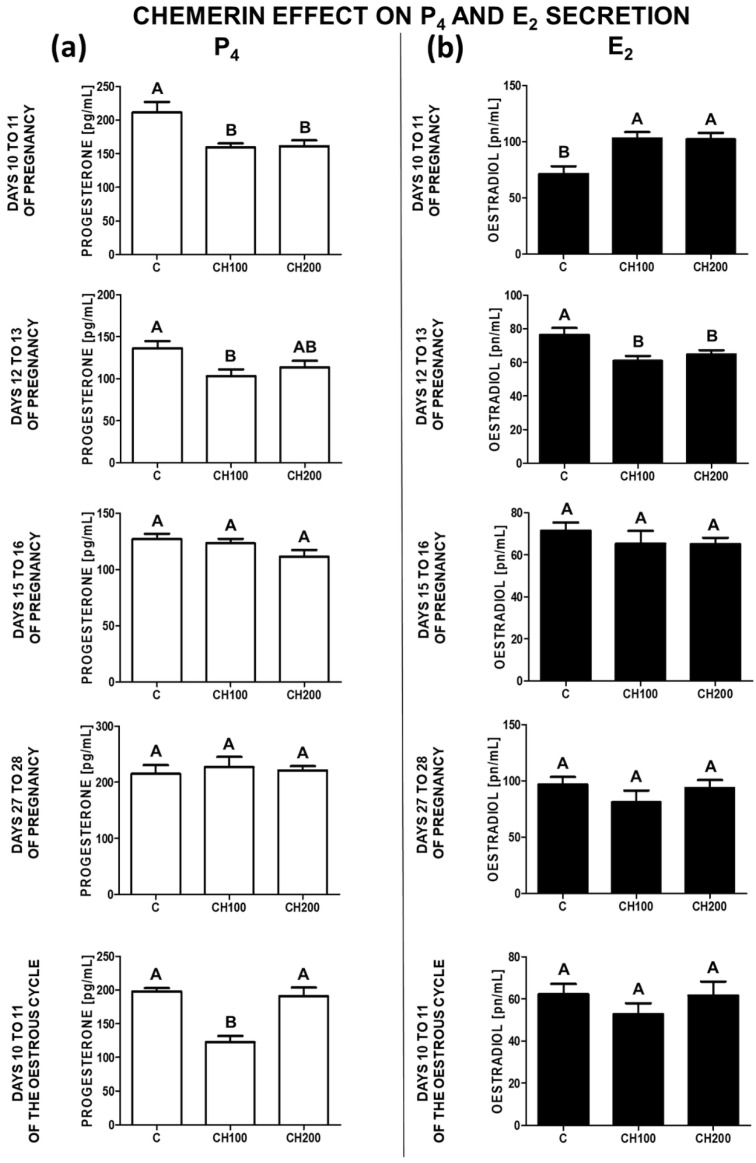
The effect of chemerin on P_4_ and E_2_ secretion. The influence of chemerin (100, 200 ng/mL) on the secretion of progesterone (P_4_; (**a**)) and oestradiol (E_2_; (**b**)) by the in-vitro-incubated endometrial tissue explants collected from pigs on days 10 to 11, 12 to 13, 15 to 16, and 27 to 28 of pregnancy, and on days 10 to 11 of the oestrous cycle. The steroid hormone concentrations in culture media were evaluated using radioimmunoassay analysis. Data are presented as the mean ± standard error of the mean (*n* = 5). Bars with different letters are significantly different at *p* < 0.05. C—control; CH100—chemerin 100 ng/mL; CH200—chemerin 200 ng/mL.

The secretion of P_4_ by the in-vitro-incubated endometrial tissues was reduced by chemerin at the doses of 100 and 200 ng/mL on days 10 to 11 of pregnancy. On days 12 to 13 of gestation and 10 to 11 of the oestrous cycle, chemerin at the dose of 100 ng/mL reduced the release of P_4_. On days 15 to 16 and 27 to 28 of pregnancy, chemerin did not affect P_4_ secretion (*p* < 0.05; Figure 1a).

On days 10 to 11 of pregnancy, the secretion of E_2_ was stimulated by chemerin at both doses. On days 12 to 13 of gestation, chemerin (100 and 200 ng/mL) reduced the secretion of E_2_. On days 15 to 28 of pregnancy and during the mid-luteal phase of the cycle, chemerin did not affect the secretion of E_2_ (*p* < 0.05; Figure 1b).

### 2.2. The Effect of Chemerin on StAR Protein Abundance in the Porcine Endometrial Tissue

**Figure 2:**
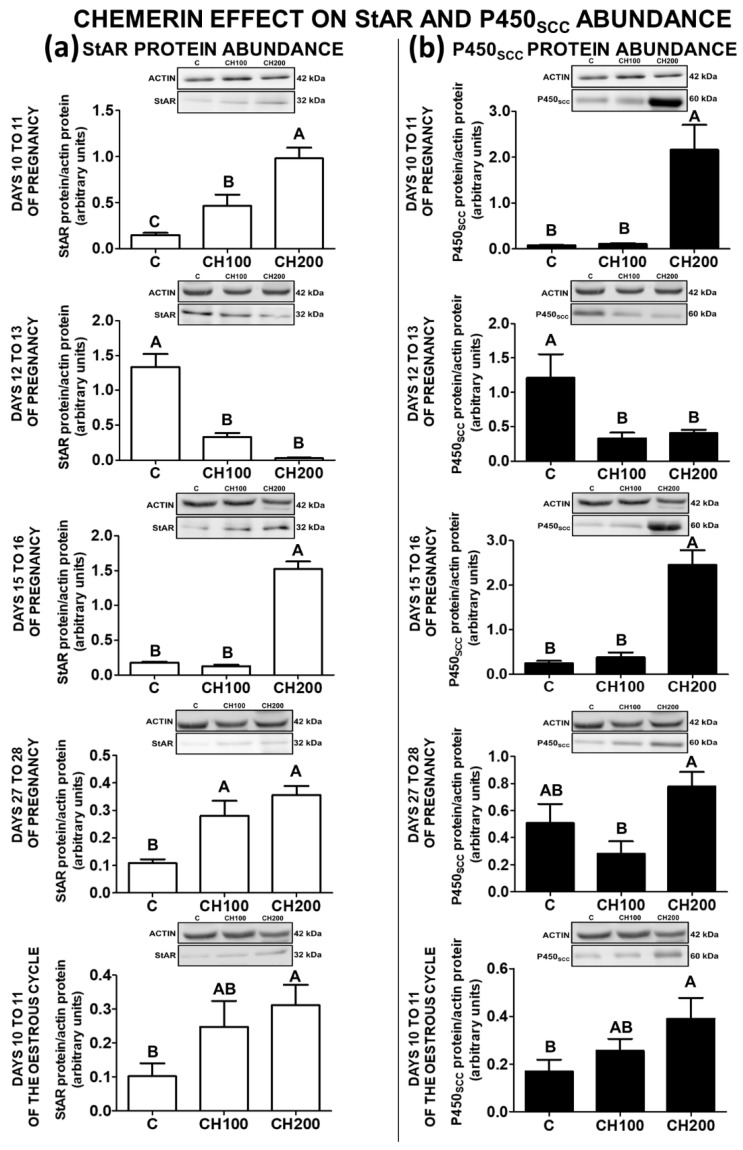
The effect of chemerin on StAR and P450_SCC_ protein abundance. The influence of chemerin (100, 200 ng/mL) on the protein abundance of steroidogenic acute regulatory protein (StAR; (**a**)) and P450 side-chain cleavage enzyme (P450_SCC_; (**b**)) in the in-vitro-incubated endometrial tissue explants collected from pigs on days 10 to 11, 12 to 13, 15 to 16, and 27 to 28 of pregnancy, and on days 10 to 11 of the oestrous cycle. The protein abundance of StAR and P450_SCC_ was evaluated using Western blot analysis. Upper panels—representative immunoblots, lower panels—densitometry analysis of target proteins’ relative content normalised with the actin protein. Data are presented as the mean ± standard error of the mean (*n* = 5). Bars with different letters are significantly different at *p* < 0.05. C—control; CH100—chemerin 100 ng/mL; CH200—chemerin 200 ng/mL.

Chemerin at the doses of 100 and 200 ng/mL enhanced the abundance of StAR protein in the porcine endometrium on days 10 to 11 and 27 to 28 of pregnancy. On days 15 to 16 of pregnancy and 10 to 11 of the oestrous cycle, StAR protein was more abundant under the influence of chemerin at the dose of 200 ng/mL. However, on days 12 to 13 of gestation, chemerin (100 and 200 ng/mL) reduced StAR protein content (*p* < 0.05; Figure 2a).

### 2.3. The Effect of Chemerin on P450_SCC_ Protein Abundance in the Porcine Endometrial Tissue

Chemerin at the dose of 200 ng/mL enhanced P450_SCC_ protein abundance in the porcine endometrial tissue on days 10 to 11 and 15 to 16 of pregnancy and during the mid-luteal phase of the cycle. Chemerin (100 and 200 ng/mL) reduced the abundance of P450_SCC_ protein in the porcine endometrium harvested on days 12 to 13 of pregnancy, whereas it had no effect on days 27 to 28 of gestation (*p* < 0.05; Figure 2b).

### 2.4. The Effect of Chemerin on 3βHSD Protein Abundance in the Porcine Endometrial Tissue

**Figure 3:**
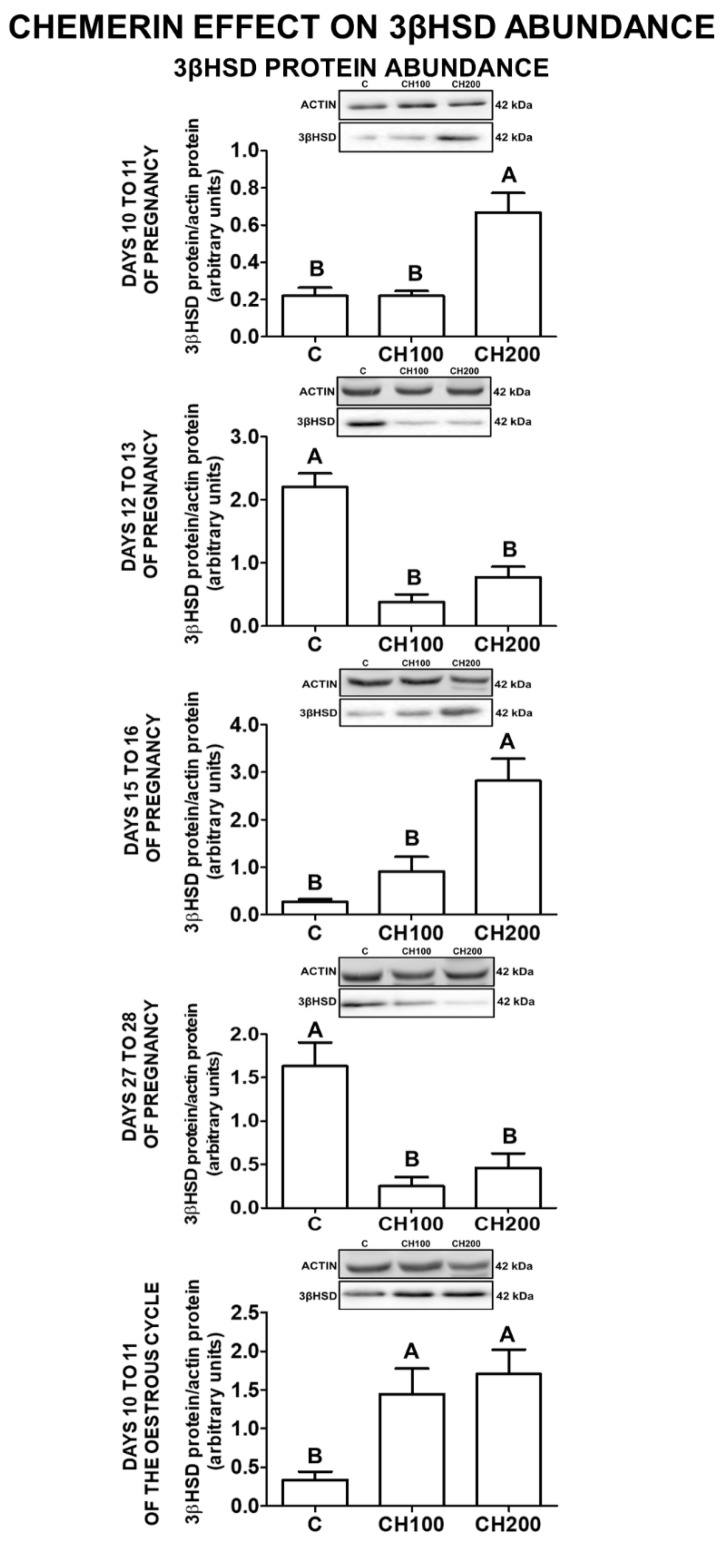
The effect of chemerin on 3βHSD protein abundance. The influence of chemerin (100, 200 ng/mL) on the protein abundance of 3β-hydroxysteroid dehydrogenase (3βHSD) in the in-vitro-incubated endometrial tissue explants collected from pigs on days 10 to 11, 12 to 13, 15 to 16, and 27 to 28 of pregnancy, and on days 10 to 11 of the oestrous cycle. The protein abundance of 3βHSD was evaluated using Western blot analysis. Upper panels—representative immunoblots, lower panels—densitometry analysis of target proteins’ relative content normalised with the actin protein. Data are presented as the mean ± standard error of the mean (*n* = 5). Bars with different letters are significantly different at *p* < 0.05. C—control; CH100—chemerin 100 ng/mL; CH200—chemerin 200 ng/mL.

The abundance of 3βHSD protein in the endometrial explants was increased under the influence of chemerin at the higher dose on days 10 to 11 and 15 to 16 of pregnancy, and at both doses used also on days 10 to 11 of the oestrous cycle. On days 12 to 13 and 27 to 28 of pregnancy, chemerin at both concentrations decreased 3βHSD protein expression (*p* < 0.05; Figure 3).

### 2.5. The Effect of Chemerin on P450_C17_ Protein Abundance in the Porcine Endometrial Tissue

**Figure 4:**
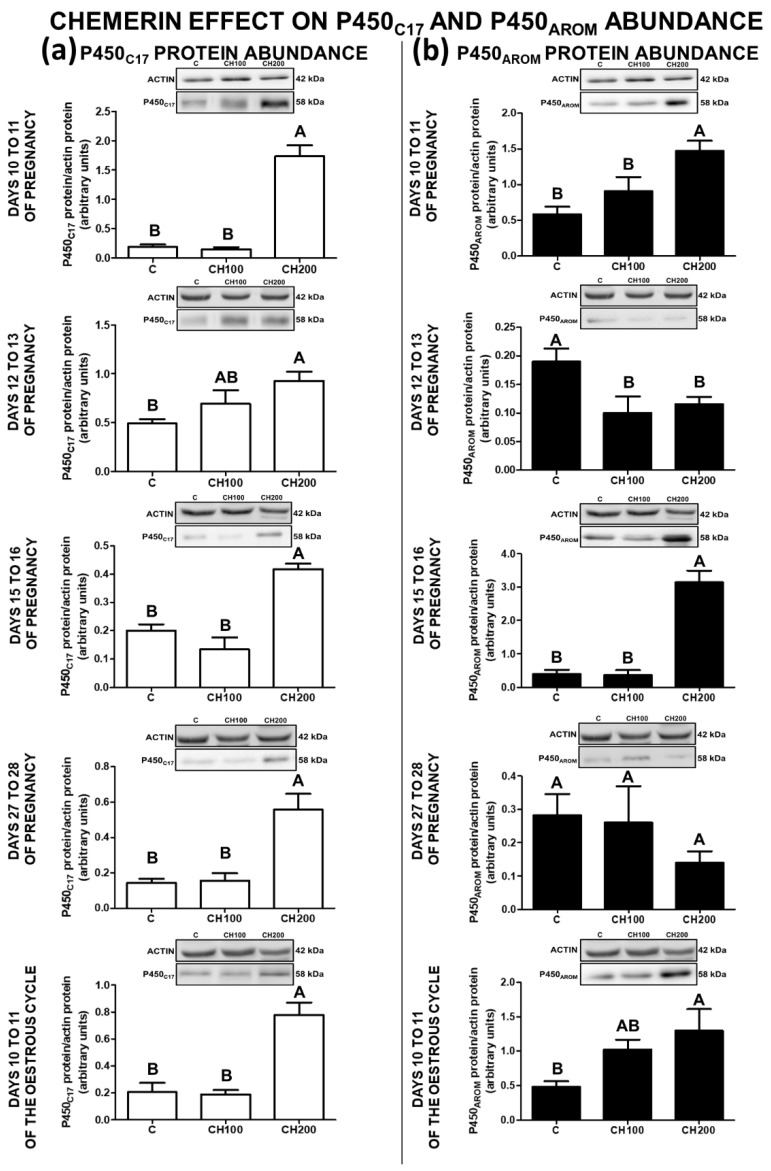
The effect of chemerin on P450_C17_ and P450_AROM_ protein abundance. The influence of chemerin (100, 200 ng/mL) on the protein abundance of cytochrome P450_C17_ (P450_C17_; (**a**)) and cytochrome P450 aromatase (P450_AROM_; (**b**)) in the in-vitro-incubated endometrial tissue explants collected from pigs on days 10 to 11, 12 to 13, 15 to 16, and 27 to 28 of pregnancy, and on days 10 to 11 of the oestrous cycle. The protein abundance of P450_C17_ and P450_AROM_ was evaluated using Western blot analysis. Upper panels—representative immunoblots, lower panels—densitometry analysis of target proteins’ relative content normalised with the actin protein. Data are presented as the mean ± standard error of the mean (*n* = 5). Bars with different letters are significantly different at *p* < 0.05. C—control; CH100—chemerin 100 ng/mL; CH200—chemerin 200 ng/mL.

The abundance of P450_C17_ protein in the endometrial explants was enhanced by chemerin at the dose of 200 ng/mL during all examined stages of early pregnancy and on days 10 to 11 of the cycle. The effect of chemerin at the dose of 100 ng/mL was negligible (*p* < 0.05; Figure 4a).

### 2.6. The Effect of Chemerin on P450_AROM_ Protein Abundance in the Porcine Endometrial Tissue

On days 10 to 11 and 15 to 16 of pregnancy, as well as during the mid-luteal phase of the oestrous cycle, chemerin at the dose of 200 ng/mL enhanced P450_AROM_ protein expression in the endometrial explants. On days 12 to 13 of gestation, the accumulation of P450_AROM_ protein in the endometrial tissue was reduced under the influence of chemerin at both tested doses. Chemerin did not affect the abundance of P450_AROM_ protein on days 27 to 28 of pregnancy (*p* < 0.05; Figure 4b).

### 2.7. The Effect of Chemerin on Erk1/2 and Akt Signalling Pathways

**Figure 5:**
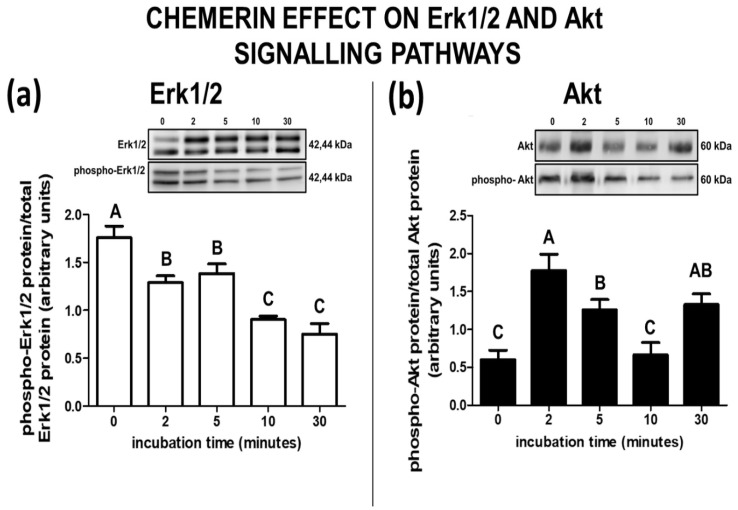
The effect of chemerin on the activation of Erk1/2 and Akt pathways. The influence of chemerin (200 ng/mL) on the activation of Erk1/2 (**a**) and Akt (**b**) signalling pathways in the in-vitro-incubated endometrial tissue explants collected from pigs on days 10 to 11 of the oestrous cycle. The total and phosphorylated Erk1/2 and Akt proteins’ abundances were evaluated using Western blot analysis. Upper panels—representative immunoblots, lower panels—densitometry analysis of phosphorylated target proteins’ relative content normalised with the total protein amounts. Data are presented as the mean ± standard error of the mean (*n* = 5). Bars with different letters are significantly different at *p* < 0.05.

Chemerin decreased the phosphorylation of Erk1/2 protein at each studied period of incubation (2, 5, 10, and 30 min) compared to the control (0 min; Figure 5a).

Chemerin activated the Akt signalling pathway in the explants after 2, 5, and 30 min of incubation compared with control samples (0 min; Figure 5b).

## 3. Discussion

The present study demonstrates, for the first time, the impact of chemerin on the secretion of P_4_ and E_2_ and abundance of StAR, P450_SCC_, P450_C17_, P450_AROM_, and 3βHSD proteins in the porcine endometrium during early pregnancy and the mid-luteal phase of the oestrous cycle. The examined enzyme proteins are essential for P_4_ and E_2_ synthesis during the steroidogenesis pathway [31]. Since the steroid hormones are not stored but constitutively released from the cells, it can be stated that chemerin affects the de novo synthesis of steroid hormones. It is well known that chemerin is involved in ovarian steroidogenesis by its action via the Erk1/2 and Akt signalling pathways [32]. In this study, we also demonstrated that chemerin activated Akt and inhibited the Erk1/2 signalling pathways in the porcine endometrium during the mid-luteal phase of the oestrous cycle, which might be related to the influence of this hormone on steroidogenesis.

This study demonstrated the effect of chemerin on the steroidogenesis processes that occur in the porcine endometrium. Our results indicate that chemerin increased E_2_ secretion on days 10 to 11 of pregnancy, whereas it decreased E_2_ release on days 12 to 13 and P_4_ production on days 10 to 13 of pregnancy and during the mid-luteal phase of the oestrous cycle. Taking into account differences related to the used tissue, the obtained data are mostly consistent with those presented by Wang et al. [29] and Reverchon et al. [32]. The authors reported that chemerin could inhibit basal and IGF-1- or FSH-induced P_4_ and E_2_ production in bovine granulosa cells (Gc) and FSH-induced P_4_ and E_2_ synthesis in the rat Gc [29,32]. In the porcine ovary, chemerin enhanced non-stimulated P_4_ production in the porcine luteal, theca interna, and Gc during the oestrous cycle, whereas it had no effect on basal E_2_ secretion by the ovarian cells [30]. The above-presented data may support the claim that, similarly to the ovaries, chemerin is engaged in the regulation of endometrial steroidogenesis.

During the establishment of pregnancy, the environmental homeostasis within the uterus, including the proper levels of steroid hormones, is essential for appropriate embryo development [33]. Oestrogens stimulate the production of PGE_2_ and change the direction of PGF_2α_ secretion from the uterine vasculature to its lumen, which protects the CL against luteolysis [34]. It is particularly well known that E_2_ of embryonic origin plays a very important role in the maternal recognition of pregnancy. Total oestrone (E_1_) and E_2_ concentrations in the uterine lumen increase around 6-fold from day 10 to day 12 of pregnancy [35]. Moreover, the amounts of total recoverable sulphates of E_1_ and E_2_ also are enhanced during this time. Concentrations of oestrogens decrease to day 15 of gestation afterwards [36]. It has been proposed that embryonic steroidogenesis may be supplied by oestrogens produced in the uterus [22]. In this study, we noted that on days 10 to 11 of pregnancy, chemerin increased the secretion of E_2_, whereas, on days 12 to 13 of gestation, the adipokine exhibited an inhibitory effect on E_2_ release by the porcine endometrium. The same pattern has been shown in the expression of 3βHSD and P450_AROM_ enzymes involved in the synthesis of E_2_. In our previous study, we demonstrated that the conceptuses express the chemerin gene and protein, and the hormone is present in the ULF of pigs, at particularly high concentrations during the maternal recognition of pregnancy [7]. It might be suggested that the enhanced secretion of E_1_ and E_2_ on days 10 to 11 of pregnancy described by Geisert et al. [36] could be related to chemerin activity in the uterus. Apart from embryos, the endometrium may also be a source of oestrogens. In the present study, the observed chemerin-dependent fluctuations in E_2_ secretion by the endometrial tissues may be a part of the mechanism responsible for creating optimal E_2_ concentrations in the uterine environment and protecting the developing embryos.

Endometrial secretory activity is under the undisputed impact of P_4_. The continuous growth and development of embryos, as well as the uterine receptivity for implantation, also depend on this steroid’s levels [35]. In pigs, treatment with exogenous P_4_ reduced levels of endogenous P_4_, indicating spontaneous luteolysis occurrence [37]. Coggins et al. [38] demonstrated that P_4_ treatment in sows during late gestation resulted in pregnancy extension; however, the foetal mortality and incidence of dystocia also increased. In the present study, we demonstrated that chemerin decreased P_4_ secretion by the endometrial tissue during the transuterine migration of embryos and maternal recognition of pregnancy. Although the previously described studies concerned the later stages of pregnancy, it can be suggested that, during early pregnancy, the effect of P_4_ at too high levels may be also detrimental or even dangerous for the proper development of the embryos. Hyper-concentrations of uterine-derived P_4_ could act as an exogenous hormone and reduce P_4_ production by the CL. This observation might relate to chemerin’s action against excessive P_4_ release from the endometrial tissue, which can thereby reduce the extra-ovarian P_4_ generation.

In this study, we found that, in many cases, the expression patterns of steroidogenic enzymes are different from P_4_ and E_2_ secretion patterns. This observation might be explained by the unavailability of a substrate to produce these steroids by the endometrial cells. One argument for such a suggestion is the negative correlation of chemerin levels in human serum with the levels of HDL [39], which is one of the sources of cholesterol. On the other hand, it could be elucidated by the influence of the hormonal milieu on the enzyme’s activity or post-translational modification of enzyme proteins. Galarreta et al. [40] and Tanaka et al. [41] noted that P_4_ downregulates the activity of 3βHSD in rat ovaries. Feedback regulation of 3βHSD under the impact of androgens was also observed in the rat testicular Leydig cells [42,43]. Differences in the effect of chemerin on enzyme protein abundance and steroid production may additionally result from the ability of both P_4_ and E_2_ to convert into the other hormones in the subsequent stages of the steroidogenesis process [31]. P_4_ can be converted into 17-OH-pregnenolone, next into androstenedione, and then into testosterone by the action of P450_C17_ and 17βHSD3, whereas E_2_ can be used by 17βHSD2 to produce E_1_ [31]. The final output of steroid hormones can be also controlled by many different factors acting together. In our RNA-seq experiments on the effects of chemerin on the porcine endometrial transcriptome during the peri-implantation period, we noted that the adipokine increased the expression of the cytochrome genes (*CYP2B22*, *CYP7B1* and *CYP1B1*) [44]. In the case of changes in the endometrial proteome, determined by LC-MS, during this period of pregnancy, we also found a stimulatory effect of chemerin on the expression of the cytochrome proteins (P4501A and P4502S1) and a reduction in the abundance of NADH-cytochrome b5 reductase 1 protein [45]. The obtained results confirm the significant influence of chemerin on cytochromes that can modify steroid hormones’ concentrations in the porcine uterus.

In the uterus, the Akt and Erk1/2 signalling pathways are involved in numerous processes. Signalling pathways such as PIK3, Erk1/2, P38 and MTOR (through activation of Akt1) cooperate in the cytoskeletal remodelling, migration, and adhesion of trophectoderm cells to mediate their attachment to the endometrium during implantation [46]. The expression of genes essential for uterine receptivity during the implantation process is caused by the influence of progestamedins and interferon tau, also via the MAPK and PI3K signalling pathways [47]. Both the Erk1/2 and Akt pathways can be induced by chemerin after its binding to CMKLR1 or GPR1 receptors [48]. The activated CMKLR1 receptor engages the MAPK cascade as the main signalling pathway [49]. The presented results show that chemerin caused a decrease in the phosphorylation of Erk1/2, which is similar to findings observed in the porcine anterior pituitary cells (APc) by Kisielewska et al. [50]. The opposite effect was noted in the human Gc, where chemerin activated the mentioned pathway [17]. Moreover, our results showing Akt activation by chemerin in the porcine endometrium are consistent with those obtained by us earlier in the APc [50] and by Reverchon et al. [17] in the human Gc. Kisielewska et al. [50] suggested that the distinct action of chemerin on the activation of signalling pathways could be explained by the different tissues and species used in the experiments. The results obtained in the presented study support this hypothesis. It has been shown that LH-induced activation of the Akt/PKB signalling pathway was associated with increased androgen production and *CYP17A1* gene expression in bovine theca cells [51]. Ortega et al. [52] showed that inhibition of the Akt induced higher P_4_ synthesis and lower androstenedione and androsterone production and *CYP17A1* gene expression in rat theca cells. Ryan et al. [53] showed that the presence of the Akt and Erk inhibitors negatively correlated with the level of E_2_ in follicular fluid and follicle growth in sheep. Moreover, the authors suggest that Akt is an important factor in FSH-dependent E_2_, P_4_, inhibin-A, and activin-A production by bovine granulosa cells [53]. This suggests that the effect of chemerin on the activation of the Akt pathway observed in this study might be related to the effect of the adipokine on P_4_ and E_2_ synthesis.

A number of studies indicate that both the Erk1/2 and Akt pathways are important in the regulation of steroid hormones’ synthesis [51,54,55]. In this study, we demonstrated that chemerin affects P_4_ and E_2_ production, as well as StAR, P450_SCC_, P450_C17_, P450_AROM_, and 3βHSD protein expression. Additionally, we indicated that chemerin rapidly activated the Akt pathway, whereas it reduced Erk1/2 phosphorylation. This may suggest that the adipokine influences steroid hormone production through these two signalling pathways. Moreover, the different expression levels of chemerin receptors (CMKLR1 and GPR1) observed during early pregnancy [7] may influence the intensity of the phosphorylation of Erk1/2 and Akt proteins. In further research, it would be worth verifying which specific messengers in the Erk1/2 and Akt signalling pathways are activated/inhibited by chemerin. It would also be valuable to investigate the effects of chemerin on other signalling pathways, such as AMPK.

## 4. Materials and Methods

### 4.1. Experimental Animals

All experiments were carried out in accordance with the Polish Act of the protection of animals used for educational or scientific purposes of 15 January 2015 [56] and directive 2010/63/EU of the European Parliament of 22 September 2010 on the protection of animals used for scientific objectives.

Tissues were collected from 25 female pigs intended for slaughter (7 to 8 months, 130 to 140 kg), according to Smolinska et al. [4]. The feeding of the animals was in line with current Polish standards, using a balanced diet (balanced protein level, the addition of exogenous amino acids, minerals, micro- and macro-elements). Fresh water was available for animals ad libitum. Gilts were split into five groups (*n* = 5 per group) as follows: days 10 to 11 (the transuterine migration of embryos), 12 to 13 (the maternal recognition of pregnancy), 15 to 16 (the beginning of implantation), and 27 to 28 (the end of implantation) of gestation and days 10 to 11 of the oestrous cycle (the mid-luteal phase; the highest activity of CL—comparable to that observed during early pregnancy). The day of the beginning of the second oestrus was considered day 0 of the oestrous cycle. Natural insemination took place on days 1 to 2 of the cycle. The retrieved uteri were transported to the laboratory in ice-cold PBS enriched with 1% antibiotic–antimycotic solution (Sigma Aldrich, St. Louis, MO, USA). The phase of the oestrous cycle was confirmed by ovarian morphology [57], whereas days of pregnancy were additionally evaluated by the presence and morphology of embryos collected from the uterine horns [58].

### 4.2. Tissue Collection and In Vitro Cultures of Endometrium

In vitro cultures of the endometrial tissue were executed according to Smolinska et al. [24] in duplicates in five independent experiments (n = 5). Explants of the endometrium harvested from the uterine horns (100 mg ± 10%) were rinsed three times in M199 medium (Sigma Aldrich, USA). Each of the obtained slices was placed in a separate glass vial containing 2 mL of phenol red-free M199 medium enriched with 5% new-born calf serum (FCS; Sigma Aldrich, USA), 0.1% BSA (MP Biomedicals, Irvine, CA, USA), and 1% antibiotic–antimycotic solution (Sigma Aldrich, USA). To determine the impact of chemerin on the release of P_4_ and E_2_ and the expression of StAR, P450_SCC_, P450_C17_, P450_AROM_, and 3βHSD proteins, after 2 h preincubation at 37 °C in 95% O_2_ and CO_2_ atmosphere, explants were treated with chemerin (100 and 200 ng/mL; RayBiotech, Norcross, GA, USA) or cultured without any treatment (control probes) under the same conditions. The doses of the adipokine used in the experiment were chosen according to our previous study [7], in which we determined the concentrations of the hormone in the porcine uterine luminal flushings (ULF). Following incubation, media and explants were collected and stored at −20 and −80 °C, respectively.

In the case of determination of chemerin’s influence on the phosphorylation of Erk1/2 and Akt, chemerin at the dose of 200 ng/mL was added to fresh phenol red-free M199 medium and then the in vitro cultures were conducted for five different incubation periods (0, 2, 5, 10, and 30 min) at 37 °C in 95% O_2_ and CO_2_ atmosphere. Then, endometrial explants were collected and stored at −80 °C.

The viability of the endometrial tissue at the end of incubation was determined by measuring lactate dehydrogenase (LDH) release. The activity of LDH in media was evaluated using a Liquick Cor-LDH kit (Cormay, Lomianki, Poland), in accordance with the manufacturer’s instructions, and then compared to LDH activity in the positive control (maximal release of LDH—total cell death obtained after homogenisation of endometrial cells). Mean LDH activity in the media after incubation was 128.7 ± 35.6 U/L (0.67% of maximal release of LDH).

### 4.3. Radioimmunoassay (RIA)

Concentrations of P_4_ and E_2_ in media were determined in accordance with methods described by Ciereszko et al. [59] and Grzesiak et al. [60]. Cross-reactivities of antisera against studied steroids have been published by Dziadkowiec et al. [61] and Szafranska et al. [62]. P_4_ levels were determined by the no-extraction assay. The extraction efficiency for the E_2_ assay was 90.67 ± 0.73%. The sensitivities of the test for P_4_ and E_2_ were 1 and 0.5 pg/mL, respectively. The range of the standard curve for P_4_ was from 1 to 1500 pg/mL, whereas, for E_2_, the range was 0.5 to 200 pg/mL. The intra- and inter-assay coefficients of variation were 1.86 ± 0.33% and 8.37% for P_4_, and 1.06 ± 0.39% and 7.74% for E_2_, respectively.

### 4.4. Protein Isolation and Western Blot

The samples of in-vitro-incubated endometrial tissues were homogenised on ice, with T-PER Tissue Protein Extraction Reagent (Thermo Fisher Scientific, Waltham, MA, USA) supplemented with protease inhibitors (Sigma Aldrich, USA). The lysates were cleared by centrifugation (10,000× *g*, 5 min, 4 °C) afterwards. The supernatants containing isolated proteins were stored at −80 °C until further analysis. The Bradford assay (Sigma Aldrich, USA) was used to determine protein concentrations in the probes. Spectrophotometric analysis of protein samples’ absorbance values was performed using an Infinite M200 Pro Reader with Tecan Magellan software (Tecan, Mannedorf, Switzerland). Western blot analysis was carried out according to Smolinska et al. [4], with modifications. Equal amounts of protein solutions (40 µg for StAR, P450_SCC_, P450_C17_, P450_AROM_, and 3βHSD or 10 µg for Erk1/2 and Akt) were used to carry out SDS-PAGE electrophoresis. Prepared samples were denatured at 95 °C for 3 min, and then proteins were separated on 12.5% SDS–polyacrylamide gels. After electrophoresis, proteins were transferred by SEMI-DRY transfer onto a 0.45 µm PVDF membrane (Merck Millipore, Kenilworth, NJ, USA). Membranes were blocked in 5% skimmed milk diluted in tris-buffered saline enriched with TWEEN-20 (TBST) and, subsequently, incubated in the primary antibody solution overnight at 4 °C. Detailed information on the primary and secondary antibodies used in the study is presented in Table 1. After incubation, the membranes were washed three times for 5 min in TBST buffer and then incubated for 1.5 h at room temperature in a solution of secondary antibody conjugated with horseradish peroxidase (HRP). Next, the membranes were incubated in Immobilon Western Chemiluminescent HRP Substrate (Merck Millipore, USA) in accordance with the manufacturer’s protocol. The received immunocomplexes were visualised using the G:Box EF Gel Documentation System with GeneSnap software (Syngene, Cambridge, UK). Appropriately sized bands were quantified densitometrically using Image Studio Lite v.5.2. software (LI-COR Biosciences, Lincoln, NE, USA). Actin was used as a reference protein to determine relative amounts of StAR, P450_SCC_, P450_C17_, P450_AROM_, and 3βHSD proteins. In the case of Erk1/2 and Akt proteins, the abundances of phosphorylated Erk1/2 and Akt proteins were normalised with total Erk1/2 or Akt protein abundance, respectively.

### 4.5. Statistical Analysis

All the analysed data had a normal distribution, as confirmed by the Shapiro–Wilk test (*p* > 0.05). All examined groups passed the Levene test for the homogeneity of variance (*p* > 0.05). Next, the data were analysed by one-way ANOVA, followed by Duncan’s post-hoc test to determine statistical differences (Statistica software; StatSoft Inc., Tulsa, OK, USA). Values of *p* < 0.05 were defined as statistically significant. All data are shown as means ± SEM from five independent observations (*n* = 5).

## 5. Conclusions

Even though the existence of uterine steroidogenesis has been known for some time, especially in the domestic pig, the control of this process is still unknown. In this study, we demonstrated that chemerin is involved in the regulation of sex steroid hormone production in the porcine endometrium. We noted that the impact of the adipokine on the release of P_4_ and E_2_, as well as on StAR and steroidogenic enzyme proteins’ abundance, was dependent on the period of early pregnancy and/or the mid-luteal phase of the oestrous cycle. This could support the hypothesis that the adipokine might represent a link between energy homeostasis and reproduction. Moreover, we demonstrated that chemerin inhibited the phosphorylation of Erk1/2 and activated the Akt signalling pathway. This might constitute evidence that these two signalling pathways are involved in chemerin’s action in the process of steroidogenesis in the porcine uterus. However, the details of this process require further research.

## Figures and Tables

**Table 1:** Specifications of antibodies used for the Western blot analysis.

Protein	Host Species	Catalogue Number and Supplier	Primary Antibody Dilution	Secondary Antibodies	Secondary Antibody Dilution
StAR	rabbit	ab96637 (Abcam, Cambridge, UK)	1:250	goat anti-rabbit (AP156P; Merck Millipore, Kenilworth, NJ, USA)	1:2000
P450_SCC_		ab175408 (Abcam, UK)	1:250		1:2000
P450_C17_		ab125022 (Abcam, UK)	1:250		1:2000
P450_AROM_		A2161 (ABclonal, Woburn, MA, USA)	1:250		1:2000
3βHSD		SAB2101086 (Sigma Aldrich, St. Louis, MO, USA)	1:200		1:2000
Erk1/2		#9102 (Cell Signaling Technology, Danvers, MA, USA)	1:1000		1:5000
phospho- Erk1/2		#9101 (Cell Signaling Technology, USA)	1:1000		1:5000
Akt		#9272 (Cell Signaling Technology, USA)	1:1000		1:5000
phospho-Akt		#9271 (Cell Signaling Technology, USA)	1:500		1:5000
Actin		A2066 (Sigma Aldrich, USA)	1:200		1:5000

P450_SCC_—P450 side-chain cleavage enzyme; P450_C17_—cytochrome P450_C17_; P450_AROM_—cytochrome P450 aromatase; 3βHSD—3β-hydroxysteroid dehydrogenase.

## Data Availability

Data available from the corresponding author on reasonable request.

## References

[1] Nagpal S., Patel S., Jacobe H., DiSepio D., Ghosn C., Malhotra M., Teng M., Duvic M., Chandraratna R.A. (1997). Tazarotene-induced gene 2 (TIG2), a novel retinoid-responsive gene in skin. J. Investig. Dermatol..

[2] Zabel B.A., Allen S.J., Kulig P., Allen J.A., Cichy J., Handel T.M., Butcher E.C. (2005). Chemerin activation by serine proteases of the coagulation, fibrinolytic, and inflammatory cascades. J. Biol. Chem..

[3] Kennedy A.J., Davenport A.P. (2018). International Union of Basic and Clinical Pharmacology CIII: Chemerin Receptors CMKLR1 (Chemerin1) and GPR1 (Chemerin2) Nomenclature, Pharmacology, and Function. Pharmacol. Rev..

[4] Smolinska N., Kiezun M., Dobrzyn K., Rytelewska E., Kisielewska K., Gudelska M., Zaobidna E., Bogus-Nowakowska K., Wyrebek J., Bors K. (2019). Expression of Chemerin and Its Receptors in the Porcine Hypothalamus and Plasma Chemerin Levels during the Oestrous Cycle and Early Pregnancy. Int J. Mol. Sci..

[5] Kisielewska K., Rytelewska E., Gudelska M., Kiezun M., Dobrzyn K., Bogus-Nowakowska K., Kaminska B., Smolinska N., Kaminski T. (2020). Relative abundance of chemerin mRNA transcript and protein in pituitaries of pigs during the estrous cycle and early pregnancy and associations with LH and FSH secretion during the estrous cycle. Anim. Reprod Sci..

[6] Rytelewska E., Kisielewska K., Kiezun M., Dobrzyn K., Gudelska M., Rak A., Dupont J., Kaminska B., Kaminski T., Smolinska N. (2020). Expression of chemerin and its receptors in the ovaries of prepubertal and mature gilts. Mol. Reprod. Dev..

[7] Gudelska M., Dobrzyn K., Kiezun M., Rytelewska E., Kisielewska K., Kaminska B., Kaminski T., Smolinska N. (2020). The expression of chemerin and its receptors (CMKLR1, GPR1, CCRL2) in the porcine uterus during the oestrous cycle and early pregnancy and in trophoblasts and conceptuses. Animal.

[8] Rourke J.L., Dranse H.J., Sinal C.J. (2013). Towards an integrative approach to understanding the role of chemerin in human health and disease. Obes. Rev..

[9] Takahashi M., Takahashi Y., Takahashi K., Zolotaryov F.N., Hong K.S., Kitazawa R., Iida K., Okimura Y., Kaji H., Kitazawa S. (2008). Chemerin enhances insulin signaling and potentiates insulin-stimulated glucose uptake in 3T3-L1 adipocytes. FEBS Lett..

[10] Bozaoglu K., Segal D., Shields K.A., Cummings N., Curran J.E., Comuzzie A.G., Mahaney M.C., Rainwater D.L., VandeBerg J.L., MacCluer J.W. (2009). Chemerin is associated with metabolic syndrome phenotypes in a Mexican-American population. J. Clin. Endocrinol. Metab..

[11] Roman A.A., Parlee S.D., Sinal C.J. (2012). Chemerin: A potential endocrine link between obesity and type 2 diabetes. Endocrine.

[12] Wittamer V., Bondue B., Guillabert A., Vassart G., Parmentier M., Communi D. (2005). Neutrophil-mediated maturation of chemerin: A link between innate and adaptive immunity. J. Immunol..

[13] Mattern A., Zellmann T., Beck-Sickinger A.G. (2014). Processing, signaling, and physiological function of chemerin. IUBMB Life..

[14] Hart R., Greaves D.R. (2010). Chemerin contributes to inflammation by promoting macrophage adhesion to VCAM-1 and fibronectin through clustering of VLA-4 and VLA-5. J. Immunol..

[15] Meder W., Wendland M., Busmann A., Kutzleb C., Spodsberg N., John H., Richter R., Schleuder D., Meyer M., Forssmann W.G. (2003). Characterization of human circulating TIG2 as a ligand for the orphan receptor ChemR23. FEBS Lett..

[16] Zhao L., Yamaguchi Y., Shen W.J., Morser J., Leung L.L.K. (2018). Dynamic and tissue-specific proteolytic processing of chemerin in obese mice. PLoS ONE.

[17] Reverchon M., Cornuau M., Ramé C., Guerif F., Royère D., Dupont J. (2012). Chemerin inhibits IGF-1-induced progesterone and estradiol secretion in human granulosa cells. Hum. Reprod..

[18] Yang Y.L., Ren L.R., Sun L.F., Huang C., Xiao T.X., Wang B.B., Chen J., Zabel B.A., Ren P., Zhang J.V. (2016). The role of GPR1 signaling in mice corpus luteum. J. Endocrinol..

[19] Carlino C., Trotta E., Stabile H., Morrone S., Bulla R., Soriani A., Iannitto M.L., Agostinis C., Mocci C., Minozzi M. (2012). Chemerin regulates NK cell accumulation and endothelial cell morphogenesis in the decidua during early pregnancy. J. Clin. Endocrinol. Metab..

[20] Goralski K.B., McCarthy T.C., Hanniman E.A., Zabel B.A., Butcher E.C., Parlee S.D., Muruganandan S., Sinal C.J. (2007). Chemerin, a novel adipokine that regulates adipogenesis and adipocyte metabolism. J. Biol. Chem..

[21] Garces M.F., Sanchez E., Acosta B.J., Angel E., Ruíz A.I., Rubio-Romero J.A., Diéguez C., Nogueiras R., Caminos J.E. (2012). Expression and regulation of chemerin during rat pregnancy. Placenta.

[22] Franczak A., Kotwica G. (2008). Secretion of estradiol-17beta by porcine endometrium and myometrium during early pregnancy and luteolysis. Theriogenology.

[23] Bazer F.W., Thatcher W.W. (1977). Theory of maternal recognition of pregnancy in swine based on estrogen controlled endocrine versus exocrine secretion of prostaglandin F2alpha by the uterine endometrium. Prostaglandins.

[24] Smolinska N., Dobrzyn K., Kiezun M., Szeszko K., Maleszka A., Kaminski T. (2016). Effect of adiponectin on the steroidogenic acute regulatory protein, P450 side chain cleavage enzyme and 3β-hydroxysteroid dehydrogenase gene expression, progesterone and androstenedione production by the porcine uterus during early pregnancy. J. Physiol. Pharmacol..

[25] Kiezun M., Smolinska N., Dobrzyn K., Szeszko K., Rytelewska E., Kaminski T. (2017). The effect of orexin A on CYP17A1 and CYP19A3 expression and on oestradiol, oestrone and testosterone secretion in the porcine uterus during early pregnancy and the oestrous cycle. Theriogenology.

[26] Kaminski T., Smolinska N., Kiezun M., Dobrzyn K., Szeszko K., Maleszka A. (2018). Effect of orexin B on CYP17A1 and CYP19A3 expression and oestradiol, oestrone and testosterone secretion in the porcine uterus during early pregnancy and the oestrous cycle. Animal.

[27] Kisielewska K., Rytelewska E., Gudelska M., Kiezun M., Dobrzyn K., Szeszko K., Bors K., Wyrebek J., Kaminski T., Smolinska N. (2019). The effect of orexin B on steroidogenic acute regulatory protein, P450 side-chain cleavage enzyme, and 3β-hydroxysteroid dehydrogenase gene expression, and progesterone and androstenedione secretion by the porcine uterus during early pregnancy and the estrous cycle. J. Anim. Sci..

[28] Rytelewska E., Kisielewska K., Gudelska M., Kiezun M., Dobrzyn K., Bors K., Wyrebek J., Kaminska B., Kaminski T., Smolinska N. (2020). The effect of orexin a on the StAR, CYP11A1 and HSD3B1 gene expression, as well as progesterone and androstenedione secretion in the porcine uterus during early pregnancy and the oestrous cycle. Theriogenology.

[29] Wang Q., Leader A., Tsang B.K. (2013). Inhibitory roles of prohibitin and chemerin in FSH-induced rat granulosa cell steroidogenesis. Endocrinology..

[30] Rytelewska E., Kiezun M., Kisielewska K., Gudelska M., Dobrzyn K., Kaminska B., Kaminski T., Smolinska N. (2021). Chemerin as a modulator of ovarian steroidogenesis in pigs: An in vitro study. Theriogenology.

[31] Bremer A.A., Miller W.L. (2014). Regulation of Steroidogenesis. Cellular Endocrinology in Health and Disease.

[32] Reverchon M., Bertoldo M.J., Ramé C., Froment P., Dupont J. (2014). CHEMERIN (RARRES2) decreases in vitro granulosa cell steroidogenesis and blocks oocyte meiotic progression in bovine species. Biol. Reprod..

[33] Ziecik A.J., Waclawik A., Kaczmarek M.M., Blitek A., Jalali B.M., Andronowska A. (2011). Mechanisms for the establishment of pregnancy in the pig. Reprod Domest Anim..

[34] Bazer F.W. (2013). Pregnancy recognition signaling mechanisms in ruminants and pigs. J. Anim. Sci. Biotechnol..

[35] Geisert R.D., Zavy M.T., Moffatt R.J., Blair R.M., Yellin T. (1990). Embryonic steroids and the establishment of pregnancy in pigs. J. Reprod. Fertil. Suppl..

[36] Geisert R.D., Brookbank J.W., Roberts R.M., Bazer F.W. (1982). Establishment of pregnancy in the pig: II. Cellular remodeling of the porcine blastocyst during elongation on day 12 of pregnancy. Biol. Reprod..

[37] Whitely J.L., Hartmann P.E., Willcox D.L., Bryant-Greenwood G.D., Greenwood F.C. (1990). Initiation of parturition and lactation in the sow: Effects of delaying parturition with medroxyprogesterone acetate. J. Endocrinol..

[38] Coggins E.G., Van Horn D., First N.L. (1977). Influence of prostaglandin F2 alpha, dexamethasone, progesterone and induced corpora lutea on porcine parturition. J. Anim. Sci..

[39] Shafer-Eggleton J., Adams-Huet B., Jialal I. (2020). Chemerin Ratios to HDL-cholesterol and Adiponectin as Biomarkers of Metabolic Syndrome. Endocr. Res..

[40] de Galarreta C.M.R., Fanjul L.F., Hsueh A.J. (1985). Progestin regulation of progesterone biosynthetic enzymes in cultured rat granulosa cells. Steroids.

[41] Tanaka N., Iwamasa J., Matsuura K., Okamura H. (1993). Effects of progesterone and anti-progesterone RU486 on ovarian 3 beta-hydroxysteroid dehydrogenase activity during ovulation in the gonadotrophin-primed immature rat. J. Reprod. Fertil..

[42] Galarreta C.M., Fanjul L.F., Adashi E.Y., Hsueh A.J. (1984). Regulation of 3 beta-hydroxysteroid dehydrogenase activity by human chorionic gonadotropin, androgens, and antiandrogens in cultured testicular cells. Ann. N. Y. Acad. Sci..

[43] Fanjul L.F., Quintana J., González J., Santana-Delgado P., Estévez F., de Galarreta C.M.R. (1992). Testicular 3 beta-hydroxysteroid dehydrogenase/delta 5-4 isomerase in the hypophysectomized rat: Effect of treatment with 5 alpha-dihydrotestosterone. J. Endocrinol..

[44] Orzechowska K., Kopij G., Paukszto L., Dobrzyn K., Kiezun M., Jastrzebski J., Kaminski T., Smolinska N. (2022). Chemerin effect on transcriptome of the porcine endometrium during implantation determined by RNA-Sequencing. Biol. Reprod..

[45] Orzechowska K., Smolinska N. (2022). The Effect of Chemerin on the Proteomic Profile of Porcine Endometrial Cells.

[46] Kim J., Erikson D.W., Burghardt R.C., Spencer T.E., Wu G., Bayless K.J., Johnson G.A., Bazer F.W. (2010). Secreted phosphoprotein 1 binds integrins to initiate multiple cell signaling pathways, including FRAP1/mTOR, to support attachment and force-generated migration of trophectoderm cells. Matrix Biol..

[47] Platanias L.C. (2005). Mechanisms of type-I- and type-II-interferon-mediated signalling. Nat. Rev. Immunol..

[48] De Henau O., Degroot G.N., Imbault V., Robert V., De Poorter C., Mcheik S., Galés C., Parmentier M., Springael J.Y. (2016). Signaling Properties of Chemerin Receptors CMKLR1, GPR1 and CCRL2. PLoS ONE.

[49] Wittamer V., Franssen J.D., Vulcano M., Mirjolet J.F., Le Poul E., Migeotte I., Brézillon S., Tyldesley R., Blanpain C., Detheux M. (2003). Specific recruitment of antigen-presenting cells by chemerin, a novel processed ligand from human inflammatory fluids. J. Exp. Med..

[50] Kisielewska K., Rytelewska E., Gudelska M., Kiezun M., Dobrzyn K., Bogus-Nowakowska K., Kaminska B., Smolinska N., Kaminski T. (2020). Expression of chemerin receptors CMKLR1, GPR1 and CCRL2 in the porcine pituitary during the oestrous cycle and early pregnancy and the effect of chemerin on MAPK/Erk1/2, Akt and AMPK signalling pathways. Theriogenology.

[51] Fukuda S., Orisaka M., Tajima K., Hattori K., Kotsuji F. (2009). Luteinizing hormone-induced Akt phosphorylation and androgen production are modulated by MAP Kinase in bovine theca cells. J. Ovarian Res..

[52] Ortega I., Villanueva J.A., Wong D.H., Cress A.B., Sokalska A., Stanley S.D., Duleba A.J. (2012). Resveratrol reduces steroidogenesis in rat ovarian theca-interstitial cells: The role of inhibition of Akt/PKB signaling pathway. Endocrinology.

[53] Ryan K.E., Glister C., Lonergan P., Martin F., Knight P.G., Evans A.C. (2008). Functional significance of the signal transduction pathways Akt and Erk in ovarian follicles: In vitro and in vivo studies in cattle and sheep. J. Ovarian Res..

[54] Tajima K., Yoshii K., Fukuda S., Orisaka M., Miyamoto K., Amsterdam A., Kotsuji F. (2005). Luteinizing hormone-induced extracellular-signal regulated kinase activation differently modulates progesterone and androstenedione production in bovine theca cells. Endocrinology.

[55] Miyoshi T., Otsuka F., Yamashita M., Inagaki K., Nakamura E., Tsukamoto N., Takeda M., Suzuki J., Makino H. (2010). Functional relationship between fibroblast growth factor-8 and bone morphogenetic proteins in regulating steroidogenesis by rat granulosa cells. Mol Cell Endocrinol..

[56] Polish Journal of Law of 2015, item 266. dziennikustaw.gov.pl. https://dziennikustaw.gov.pl/DU/2015/266.

[57] Akins E.L., Morrissette M.C. (1968). Gross ovarian changes during estrous cycle of swine. Am. J. Vet. Res..

[58] Anderson L.L. (1978). Growth, protein content and distribution of early pig embryos. Anat. Rec..

[59] Ciereszko R.E., Petroff B.K., Ottobre A.C., Guan Z., Stokes B.T., Ottobre J.S. (1998). Assessment of the mechanism by which prolactin stimulates progesterone production by early corpora lutea of pigs. J. Endocrinol..

[60] Grzesiak M., Knapczyk-Stwora K., Ciereszko R.E., Wieciech I., Slomczynska M. (2014). Alterations in luteal production of androstenedione, testosterone, and estrone, but not estradiol, during mid- and late pregnancy in pigs: Effects of androgen deficiency. Theriogenology.

[61] Dziadkowiec I., Danilczyk U., Rembiesa R. (1982). Biosynteza progesteronu w łlozysku szczura [Biosynthesis of progesterone in the rat placenta]. Endokrynol. Pol..

[62] Szafrańska B., Ziecik A., Okrasa S. (2002). Primary antisera against selected steroids or proteins and secondary antisera against gamma-globulins--an available tool for studies of reproductive processes. Reprod. Biol..

